# Liver Mass Caused by *Mycobacterium fortuitum*: A Rare Case Diagnosed by EUS-FNA

**DOI:** 10.14309/crj.0000000000001083

**Published:** 2023-07-07

**Authors:** Yue-Sai Jao, Lemuel Martinez, Victor J. Carlo, Carlos G. Micames

**Affiliations:** 1 Fellowship Program, University of Puerto Rico School of Medicine Gastroenterology, San Juan, Puerto Rico; 2 Infectious Disease Department, Manati Medical Center, Manati, Puerto Rico; 3 Pathology Department, Puerto Rico Pathology, San Juan, Puerto Rico; 4 Gastroenterology Department, Bella Vista Hospital, Mayaguez, Puerto Rico

**Keywords:** atypical mycobacteria, *Mycobacterium fortuitum*, liver mass, endoscopic ultrasound (EUS), fine needle aspiration (FNA)

## Abstract

*Mycobacterium fortuitum* is associated with skin and soft-tissue infections, yet isolated liver involvement is rare. A 67-year-old asymptomatic man was referred for endoscopic ultrasound (EUS) to evaluate a gastric lesion and an incidental liver mass. EUS revealed a heterogeneous liver mass that was sampled. Pathology revealed necrotic granulomatous inflammation and positive acid-fast bacilli stain with *M. fortuitum* deoxyribonucleic acid. Levofloxacin plus trimethoprim and sulfamethoxazole for 3 months were used for complete resolution of liver lesion. Isolated nontuberculous liver involvement is uncommon. We report the first case of a liver mass caused by *M. fortuitum* diagnosed by EUS-fine needle aspiration.

## INTRODUCTION

*Mycobacterium fortuitum* is a type of nontuberculous mycobacteria belonging to a group classified as rapidly growing mycobacteria and *Mycobacterium abscessus* and *Mycobacterium chelonae*. These nontuberculous mycobacteria can be found in soil and aquatic environments. *Mycobacterium fortuitum* is an opportunistic pathogen characterized by biofilm formation as attributed to known antibiotic resistance.^1^ *M. fortuitum* is predominantly associated with skin, soft-tissue, and surgical wound infections. Nosocomial infections such as catheter-related sepsis, prosthetic arthroplasty, and pacemaker infection have been fairly described. Disseminated diseases such as pneumonia, endocarditis, and meningitis can also occur.^2^ Isolated liver involvement by atypical mycobacterial infection is exceedingly rare. Here, we describe a case of *M. fortuitum* presenting as an asymptomatic liver mass.

## CASE REPORT

**Figure 1.**
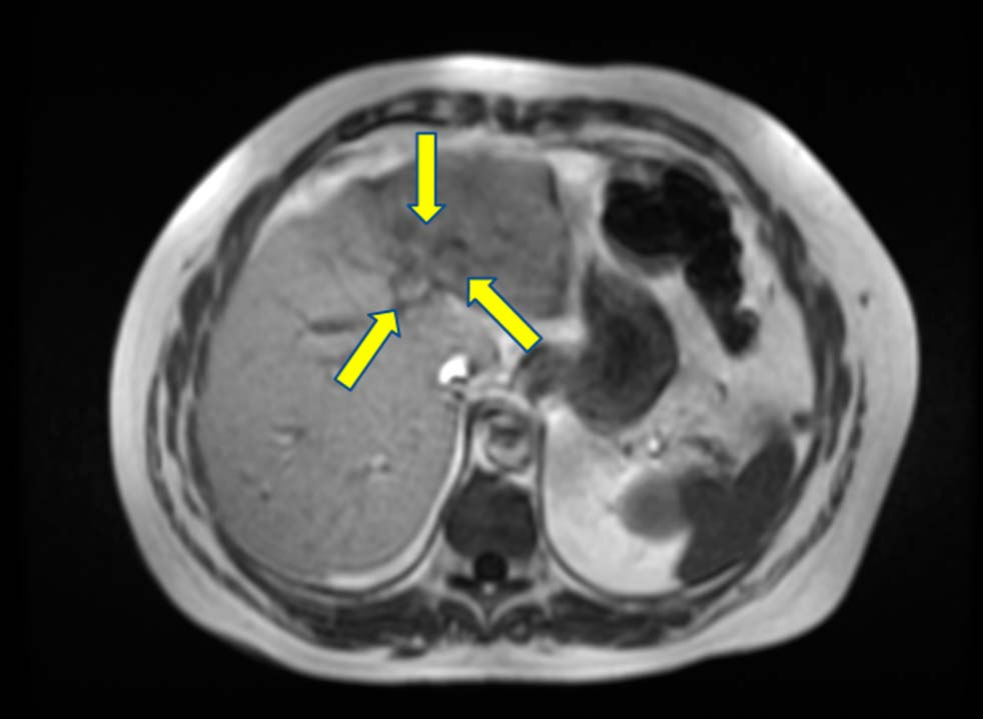
T1-weighted imaging of the liver mass.

**Figure 2.**
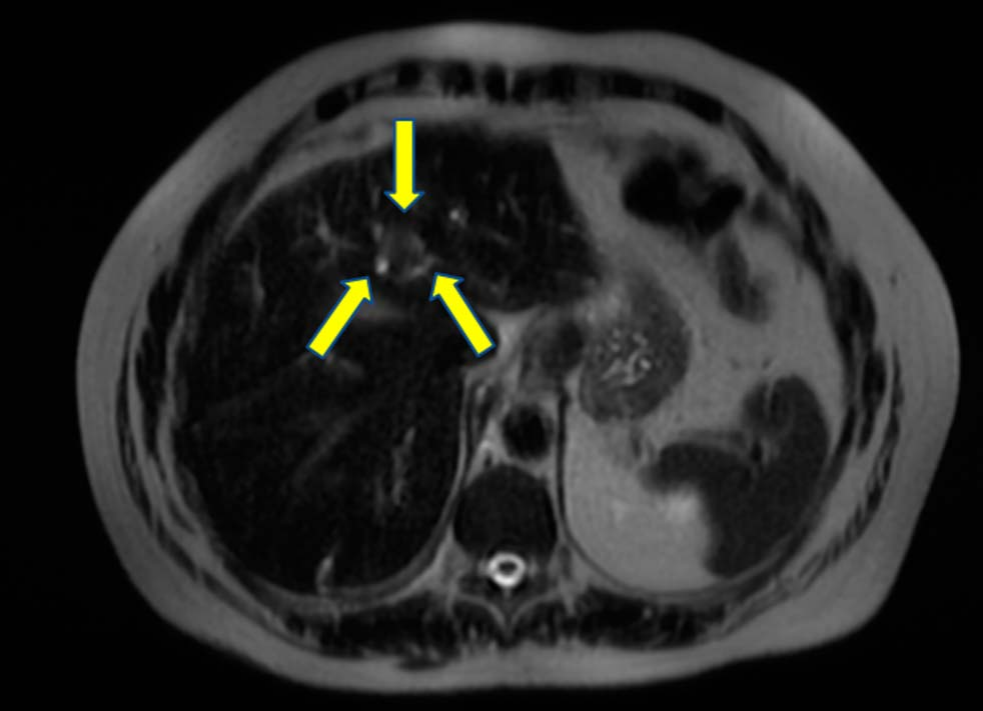
T2-weighted imaging of the liver mass.

**Figure 3.**
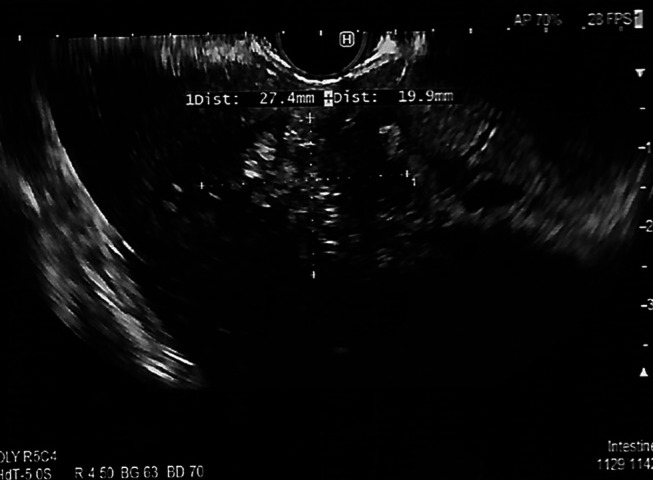
Endoscopic ultrasound of the liver mass.

**Figure 4.**
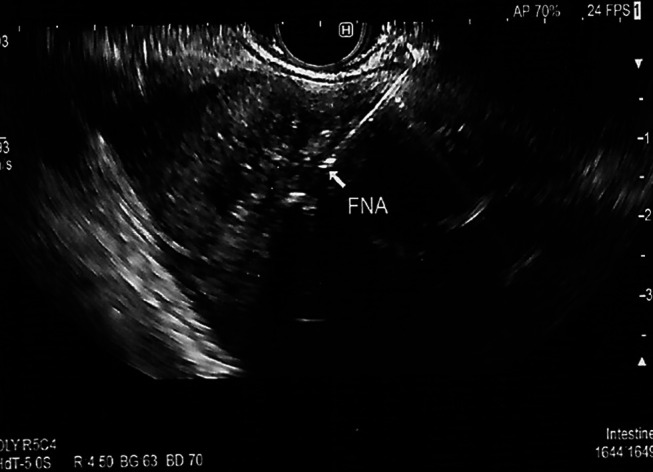
Endoscopic ultrasound-fine needle aspiration of the liver mass.

**Figure 5.**
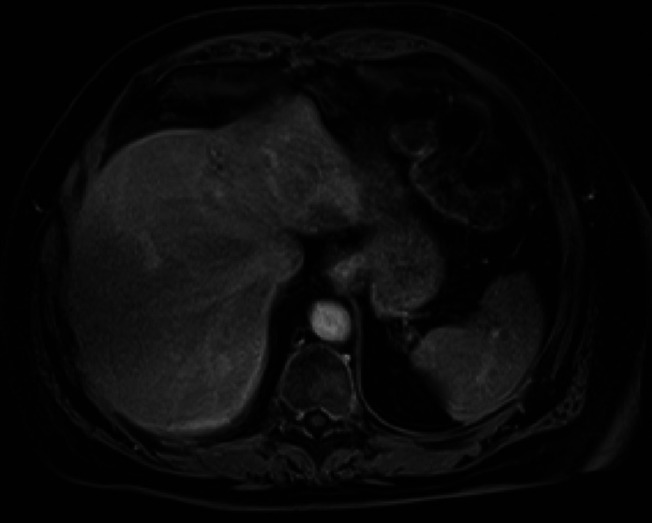
T1-weighted imaging of the liver posttreatment of *M. fortuitum.*

**Figure 6.**
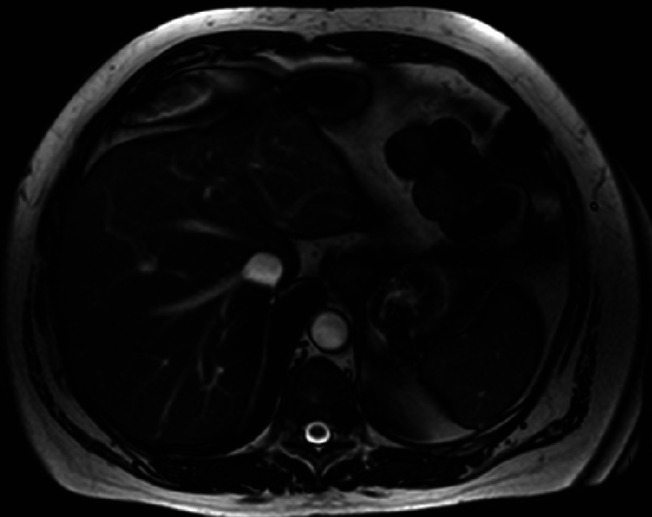
T2-weighted imaging of the liver posttreatment of *M. fortuitum.*

A 67-year-old man was referred for endoscopic ultrasound (EUS) to evaluate a gastric body subepithelial lesion (SEL) and a 3 cm incidental liver mass described as a heterogenous enhancing liver lesion in the left liver lobe segment #2 and #3 (Figures 1 and 2). He was asymptomatic. Percutaneous computed tomography (CT)-guided liver biopsy was performed, but pathologic results were nondiagnostic. The patient was thus referred for EUS to evaluate the gastric SEL and determine whether the liver mass represented metastasis. A 14 by 4 mm elongated intramural hypoechoic mass arising from the muscularis propria was identified. A heterogeneous mass with cystic spaces and echogenic foci in the left liver lobe measuring 27 by 20 mm was sampled using a 22 g FNA needle (Figures 3 and 4). Pathology revealed granulomatous inflammation with necrosis. An acid fast bacilli stain was positive for acid-fast bacilli. Polymerase chain reaction analysis of the specimen detected *M. fortuitum* deoxyribonucleic acid. The patient was treated with levofloxacin 750 mg daily plus trimethoprim and sulfamethoxazole (Septra Double Strength) 800–160 mg twice daily. Follow-up contrast-enhanced MRI revealed complete resolution of the liver lesion after 3 months of antibiotics (Figures 5 and 6).

## DISCUSSION

The liver mass was initially believed to be evidence of a metastatic primary gastric tumor despite an isolated hepatic lesion found in the setting of a subepithelial lesion that is commonly found in routine examinations and usually benign in nature.^3^ Initial percutaneous CT-guided liver biopsy was nondiagnostic and falsely negative in retrospect, either from sampling error or from insufficient tissue acquisition. Acid-fast staining is not included in routine histopathology workup unless infectious etiology is considered in the differential diagnosis. A study revealed a diagnostic accuracy of 86% CT-guided liver biopsy of small focal liver lesions: 3 cm.^4^ Percutaneous liver biopsy suffers shortcomings in diagnostic accuracy because of the requirements of a large sample, operator's skill, and sampling error.^5^ EUS findings showed a gastric mass extending to muscularis propria highly suggestive of a gastrointestinal stromal tumor or leiomyoma,^3^ the latter is at low risk for malignant transformation.^6^ Gastrointestinal stromal tumor has the potential to metastasize, but, in our case, the lesion was in the stomach, was small in size (less than 2 cm) with an asymptomatic presentation; therefore, this SEL was a low risk for malignancy and therefore managed with annual endoscopic surveillance.^7^ *Mycobacterium fortuitum* has not been previously associated with liver involvement. By contrast, hepatic tuberculosis can emerge in a variety of presentations that include miliary tuberculosis, tuberculoma, pseudotumor, and abscess.

Abdominal MRI showed hyperintensity on T2-weighted imaging sharing similarities with radiologic findings of a hepatic tuberculoma or abscess.^8^ EUS showed no well-defined fluid collections but rather a hyperechoic mass in favor of a hepatic tuberculoma. Like a hepatic tuberculoma, this hepatic mass resolved with antimicrobial agents and did not require drainage. Few publications describe isolated liver involvement by an atypical mycobacterial infection,^9,10^ but none are associated with *M. fortuitum*.

Isolated liver involvement by nontuberculous mycobacteria is uncommon, usually asymptomatic, and its diagnosis can easily be missed or mistaken. We report the first case of a liver mass caused by *M. fortuitum* diagnosed by EUS-fine needle aspiration (FNA). Precise and accurate recognition by a minimally invasive technique such as EUS-FNA resulted in prompt treatment and resolution of the disease.

## Disclosures

Author contributions: Y-S. Jao: conceptualization, investigation, writing, review & editing, and project administration. L. Martinez and VJ Carlo: conceptualization, investigation, and validation. C. Micames: conceptualization, investigation, writing, review & editing, and supervision.

Declaration of competing interest or financial support. Dr. Lemuel Martinez discloses being a speaker for Cubist Pharmaceuticals. Dr. Carlos G Micames has served as a consultant for Boston Scientific. The remaining authors have no financial interests disclosed related to this article.

Notice of prior presentation of the case report at a professional meeting: The case report was presented on October 24, 2022, at the ACG national conference meeting in Charlotte, NC.

Informed consent was obtained for this case report.
